# Current genetic models for studying congenital heart diseases: Advantages and disadvantages

**DOI:** 10.6026/973206300200415

**Published:** 2024-05-31

**Authors:** Ayat Shorbaji, Peter Natesan Pushparaj, Sherin Bakhashab, Ayat B Al-Ghafari, Rana R Al-Rasheed, Loubna Siraj Mira, Mohammad Abdullah Basabrain, Majed Alsulami, Isam M Abu Zeid, Muhammad Imran Naseer, Mahmood Rasool

**Affiliations:** 1 Biochemistry Department, King Abdulaziz University, Jeddah, Saudi Arabia; 2 Center of Excellence in Genomic Medicine Research, Department of Medical Laboratory Technology, Faculty of Applied Medical Sciences, King Abdulaziz University, Jeddah, Saudi Arabia; 3 Experimental Biochemistry Unit, King Fahd Medical Research Center, King Abdulaziz University, Jeddah, Saudi Arabia; 4 Experimental Biochemistry Unit, King Fahad research Center, King Abdulaziz University, Jeddah, Saudi Arabia; 5 Department of Biological Sciences, Faculty of Science, King Abdulaziz University, Jeddah, Saudi Arabia

**Keywords:** Congenital heart disease, *in vivo* models, *In vitro* models, genetic mutations

## Abstract

Congenital heart disease (CHD) encompasses a diverse range of structural and functional anomalies that affect the heart and the major blood vessels. Epidemiological studies have documented a global increase in CHD prevalence, which can be attributed to advancements in diagnostic technologies. Extensive research has identified a plethora of CHD-related genes, providing insights into the biochemical pathways and molecular mechanisms underlying this pathological state. In this review, we discuss the advantages and challenges of various *In vitro* and *in vivo* CHD models, including primates, canines, Xenopus frogs, rabbits, chicks, mice, Drosophila, zebrafish, and induced pluripotent stem cells (iPSCs). Primates are closely related to humans but are rare and expensive. Canine models are costly but structurally comparable to humans. Xenopus frogs are advantageous because of their generation of many embryos, ease of genetic modification, and cardiac similarity. Rabbits mimic human physiology but are challenging to genetically control. Chicks are inexpensive and simple to handle; however, cardiac events can vary among humans. Mice differ physiologically, while being evolutionarily close and well-resourced. Drosophila has genes similar to those of humans but different heart structures. Zebrafish have several advantages, including high gene conservation in humans and physiological cardiac similarities but limitations in cross-reactivity with mammalian antibodies, gene duplication, and limited embryonic stem cells for reverse genetic methods. iPSCs have the potential for gene editing, but face challenges in terms of 2D structure and genomic stability. CRISPR-Cas9 allows for genetic correction but requires high technical skills and resources. These models have provided valuable knowledge regarding cardiac development, disease simulation, and the verification of genetic factors. This review highlights the distinct features of various models with respect to their biological characteristics, vulnerability to developing specific heart diseases, approaches employed to induce particular conditions, and the comparability of these species to humans. Therefore, the selection of appropriate models is based on research objectives, ultimately leading to an enhanced comprehension of disease pathology and therapy.

## Background:

**Figure 1:**
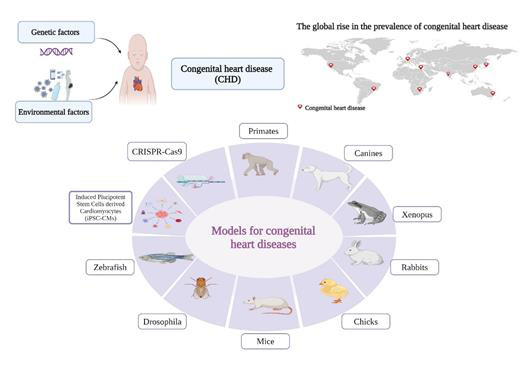
*In vitro* and in vivo models to study the congenital heart diseases.

Structural or functional abnormalities in the heart or major vessels at birth are characteristic of congenital heart disease (CHD). These anomalies are attributed to genetic variation, environmental influences, or a combination of both elements [1]. The most common type of birth defect is congenital heart defect [2]. The prevalence of CHD is on the rise, reaching 9.41 per 1000 live births during the previous 15 years, signifying a substantial escalation in the global impact of CHD [3]. Various factors influence documented birth prevalence, including the definition of CHD, diagnostic capacity, screening and detection methods, and administrative considerations, such as diagnosis registration. Giang *et al.* identified ethnicity and genetic, environmental, and socioeconomic factors as potential additional variables influencing birth prevalence [4]. A recent study documented geographical disparities in the prevalence of CHD, with the lowest and highest rates in Africa and Asia, respectively [3]. Congenital cardiac defects can be classified into various categories, which can be employed to highlight the fundamental anatomical and pathophysiological aspects. These defects can be classified into four main categories: CHD characterized by a shunt between the systemic and pulmonary circulation, CHD associated with left or right heart issues, CHDs involving the aberrant origin of the major arteries, and CHD accompanied by other coexisting disorders [5]. CHD continues to be a significant contributor to both mortality and morbidity among individuals across their lifespan, including children and adults [6]. Congenital arrhythmias can be potentially life-threatening and lead to abrupt cardiac death [7]. CHD can be hereditary or non-genetic. Despite decades of international efforts to address these factors, the number of nongenetic causes of CHD is still expanding and changing. Dioxins, pesticides, and polychlorinated biphenyls are environmental factors. In addition, CHD can be caused by maternal exposure to alcohol, isotretinoin, thalidomide, and antiseizure medications. Other CHD risk factors include taking antiretroviral medications and obesity associated with diabetes mellitus and hypercholesterolemia [8]. Evidence supporting genetic underpinnings of CHD is multifaceted. A higher concordance in monozygotic twins than in dizygotic twins indicates a genetic predisposition, even as twinning itself emerges as a modest risk factor for CHD [9]. The recurrence risk among siblings for related and discordant forms of CHD further underscores genetic influences. A minority of rare Mendelian forms of CHD offer crucial insights into conditions, such as atrial septal defects, heterotaxy, mitral valve prolapse, and bicuspid aortic valve [9]. Intriguingly, CHD cases within families without a history of CHD significantly contribute to de novo genetic events including chromosomal abnormalities, copy number variants (CNVs), and point mutations. A noteworthy aspect of CHD is its increased prevalence in populations characterized by high consanguinity, implying the involvement of recessive genetic factors. Genetic factors play a significant role in the etiology of CHD considering the potential interplay between genetics and environmental triggers [9]. The accurate determination of the genetic factors responsible for heart abnormalities is challenging. This is primarily due to the complex nature of the genetic network that governs heart development [10]. As mentioned in the Introduction, the genetics of CHD are heterogeneous [11]. According to epidemiological research, the prevalence of single-gene disorders in individuals with CHD as part of a syndrome ranges from 3% to 5%. Moreover, gross chromosomal aberrations or aneuploidy are detected in approximately 8-10% of individuals with CHD as part of a syndrome, whereas pathogenic CNVs are observed in 3-25% of the same population. Among individuals with isolated CHD, the prevalence of pathogenic CNVs ranged from 3% to 10%. [12]. Extensive genetic analysis of CHD using next-generation sequencing (NGS) indicated that approximately 8% and 2% of the cases can be attributed to de novo autosomal dominant and inherited autosomal recessive variations, respectively [13]. Although diligent endeavours have been made in this field, the precise genetic pathways underlying CHD remain inadequately understood, and an estimated 55% of individuals affected by CHD do not have a genetic diagnosis [14]. Yasuhara and Garg summarized non-syndromic (Table 1) and syndromic (Table 2) CHD-associated genes [15]. Researchers have developed several models to understand the genetic factors associated with CHD and identify the genes responsible for its occurrence. In this review, we aimed to highlight the most common *in vivo* and *In vitro* models, and how these models were employed to validate the causative genes of CHD in humans (Figure 1).

## CHD Gene Modeling Systems:

## Primates:

The protein-coding sequences of chimps are similar (99.1 %) to those of humans, whereas approximately two-thirds of the amino acid sequences are identical, making them good candidates for modeling CHD genetics [16]. In 2023, Gao *et al.* obtained whole genome sequencing data for 809 individuals from 233 primate species and used a deep learning classifier trained on 4.3 million common primate missense variants to predict variant pathogenicity in humans. The similarity between primates and humans enables them to determine the effects of human genetic variants systematically. In addition, the same study distinguished de novo missense mutations in 2,871 CHD patients from de novo missense those in 2,555 healthy controls [17]. Chimps have a number of benefits for genetic studies: long-term maintenance of constant environmental conditions increases the ability to detect genetic effects, sequential application of various environmental conditions to individuals can characterize genotype-environment interactions, generation of complex pedigrees, which are much more effective for genetic analysis than commonly available human pedigrees, and prospective testing of genetic hypotheses through selective mating [18]. Despite this potential, the use of primates, especially chimps, as models is still limited owing to age-old limitations in availability and cost [18].

## Canines:

Canine families and domestic dogs can have more than 450 diseases, approximately 360 of which are similar to human diseases. Genetic studies in dogs are theoretically easier and more straightforward than those conducted in complex populations, providing statistical advantages equal to those of studies performed in isolated human populations [19]. Dogs and humans share many similarities in the structure and composition of their heart. Dogs are more similar to humans than mice, rats, or rabbits in terms of heart rate, body weight, and heart weight. This means that canines can be assessed for contractility using procedures primarily designed for human hearts owing to their similar size [20]. A study of 700 dogs with CHD found that the type and occurrence of defects in dogs and humans are similar. Certain breeds show a higher incidence of specific anomalies, which can be used as models for studies on genetic and environmental factors [21]. The discovery of a new missense variant in the transient tachypnea of the newborn (TTN) gene, which contributes to CHD in Doberman pinscher dogs, can be compared with its variants in humans, as TTN variants contribute to hypertrophic and dilated cardiomyopathies in humans [22]. Nevertheless, the expenses associated with conducting long-term chronic investigations in disease states, including initial purchase costs and daily charges, may pose significant barriers [23]. Additionally, it is necessary to obtain the required approval to conduct research on these species [20].

## Xenopus:

Xenopus frogs, notably Xenopus laevis and Xenopus tropicalis, offer versatile and efficient *in vivo* systems for investigating human diseases. These species are valuable models with unique strengths, which can be tailored to specific research approaches. Although Xenopus species possess distinct attributes, they share key experimental advantages that have made them pivotal in embryology. The ability to breed Xenopus year-round, yielding substantial clutch sizes of up to 2000 eggs per frog per day, coupled with straightforward *In vitro* fertilization, ensures a continuous supply of developmentally synchronized embryos. These embryos undergo external development, rendering them accessible for microinjection-based genetic manipulation. With its uncomplicated husbandry, Xenopus has emerged as an affordable and practical model for large-scale experiments, including screening and characterizing candidate genes related to human diseases. The frog model has been instrumental in employing genetic knockdown approaches such as morpholino (MO)s and mRNA overexpression of well-known disease-associated genes in embryonic development [24]. Moreover, the cardiac morphology of Xenopus has a greater resemblance to that of humans than that of fish. For example, Xenopus shares certain characteristics with humans, including the atrial septation. In addition, Xenopus possesses a comparatively compact diploid genome, measuring approximately 1.5 GB in size. This compact genome retains a significant degree of synteny with the human genome, thereby facilitating the identification of orthologous genes. Furthermore, the capacity to generate a substantial number of embryos and the lack of recent genome duplications has enhanced the feasibility of employing MO knockdown technology for screening purposes [24]. Xenopus continues to illuminate the complexities of CHD, contributing to advancements in our understanding of its critical conditions. The genes that were characterized and validated using the Xenopus model are summarized in Table 3 [25].

Although Xenopus is widely recognized as a valuable model organism, it has several limitations that impede its utility in genetic studies. Initially, it was noteworthy that X. laevis could be classified as a pseudo-tetraploid because of an extra genome duplication event that occurred approximately 30 million years ago, which distinguished it from other vertebrates. In addition to the increased genome size associated with pseudotetraploidy, the likelihood of successful mutagenesis screening was diminished because of the functional redundancy observed among closely related paralogous genes. One notable drawback of X. laevis is its comparatively long generation time, typically spanning 1-2 years. Consequently, the process of generating stable transgenic lines is hindered at a slow pace [26].

## Rabbits:

Rabbits (*Oryctolagus cuniculus*) exhibit cellular electrophysiology and Ca^2+^ transport that resembles those observed in humans to a greater extent than in rats or mice. Alterations in ion channels and calcium transporters are anticipated to directly affect contractile function and the occurrence of arrhythmias, rendering them of considerable importance in the study of heart failure (HF) and arrhythmias. The ventricular action potentials (APs) of mice and rats are characterized by their brevity and the absence of the prominent AP plateau phase observed in humans, rabbits, and larger mammals. Animal transgenesis has led to significant advancements in the replication of human cardiac diseases in rabbits [27]. Significant progress has been made in transgenic research with the successful creation of an initial Short QT syndrome (SQT1) transgenic rabbit model [28]. This model effectively replicated the phenotypic characteristics of the corresponding human disease across several levels, including ion current, cellular, tissue, whole-heart, and *in vivo* simulations, specifically in the ventricles and atria. The model overexpresses a disease-specific human mutation (KCNH2/HERG-N588K5) under the control of the rabbit β-myosin-heavy-chain-promoter in the heart without concomitant structural alterations, and thus has no confounding effects on electrical features and arrhythmogenesis [28]. Despite this advancement, we should consider that the results may not be transferred across species, and more funds are needed to create transgenic control rabbits with inert genes [29]. Other disadvantages include lower efficacy of genetic manipulation, lower reproduction rates, and relatively higher housing/breeding costs [27].

## Chicken:

Chicken embryos have been used as models to study cell migration, tissue patterns, tissue symmetry, vasculogenesis, and specific organ system biology, including cardiac morphogenesis, because of their advantages such as ease of in ovo visualization, ease of manipulation, low cost, well-characterized properties, and amenability to new molecular tools [30]. Although chicks may not be as genetically tractable as mice for simulating syndromic CHD, they remain a useful model for studying structural cardiac diseases. However, it may not always be possible to accurately replicate abnormal cardiogenesis in chicks for human CHD patients because of the differences in certain cardiac events between chicks and humans, such as the development of the septum secundum and pharyngeal arch artery system [31].

## Mice:

Cardiovascular disease (CVD) is best studied in mouse models, as it has a four-chambered heart and is evolutionarily more closely related to humans than flies or zebrafish [32]. Studies in mice have shown that more than 500 mutated genes contribute to heart defects [33]. Among these abnormalities, the genetic interaction between Tbx5 and Mef2c causes ventricular septation defects in transgenic mice [34]. A comprehensive understanding of the genes, mutations, and underlying mechanisms responsible for the onset and progression of hereditary and de novo CHD in humans remains incomplete. Spielmann *et al.*, 2022 screened 3,894 single-gene-null mouse lines for structural and functional cardiac abnormalities and identified approximately 705 lines with ventricular dilation, cardiac arrhythmia, and/or myocardial hypertrophy [35]. The validated genes are listed in Table 4 [36].

Hao *et al.* identified a novel gene, WDR62, as a susceptibility gene for CHD with a high variant frequency because it plays a role in spindle assembly and cell cycle pathways of cardiomyocytes, which can affect cardiac development [37]. Although animal models provide the most accurate representation of the *in vivo* environment, it is important to note that animals differ from humans in terms of their physiology and genomics. Therefore, these factors may not always be clinically relevant [38]. The challenge of applying findings from animal studies to humans is due to the differences between species and variations across species. Therefore, the validity of preclinical animal studies is essential for extrapolation. External validity includes controllable factors, such as animal sample representativeness, relevance of animal models to therapy, and unchangeable features, such as differences between animal and human species [39].

## Drosophila:

The fruit fly shares approximately 75% of disease-associated genes with humans, making it a reliable model organism for studying a diverse range of human illnesses. Genetic makeup of the fruit fly provides valuable insights into disease pathways, from neurological and endocrine issues to muscular and cardiac ailments. Using Drosophila genetics, researchers can uncover the role of genes and pathways in channelopathies and cardiomyopathies, understand how protein mutations initiate signaling events that cause cardiac remodeling, verify DNA variants linked to cardiovascular disorders, and screen for potential drugs for innovative therapies [40]. Despite the simpler heart structure of flies and the large evolutionary gap between flies and humans, the fly heart shares many structural and functional similarities with the human heart during its early development. Combined with available genetic tools and resources, the fly heart has become a valuable model system for studying human cardiac diseases. NKX2.5 (known as tinman (Tin) in flies), a key gene in heart development, is also a genetic hotspot for variants linked to CHD. Genomic research has revealed that many patients with CHD or cardiomyopathy are likely to have a polygenic cause, and several polygenic fly models of cardiac diseases have been successfully generated, demonstrating their feasibility [41]. Drosophila have been used as a model to simulate a specific variant of uncertain significance in the human cardiogenic gene Nkx2.5. Scientists have identified genetic variations that require functional experimentation to determine their clinical relevance by sequencing the human genome samples. The Drosophila model has been employed to investigate mutations with uncertain implications in Nkx2.5 associated with CHD in humans [42]. An R321N allele of the Nkx2.5 ortholog tin was produced to simulate a human K158N mutation. The functionality of this allele has been assessed both *In vitro* and *in vivo*. *In vitro* experiments revealed that the R321N Tin isoform exhibited limited binding affinity towards DNA and showed a deficiency in its ability to activate a Tin-dependent enhancer in tissue culture. The mutant Tin variant exhibited a notable decrease in its interaction with Dorsocross1, a Tbox cardiac factor in Drosophila. The R321N allele was generated using the CRISPR/Cas9 system. Homozygotes carrying this allele exhibited viability and normal heart specifications. However, they displayed impairments in the differentiation of the adult heart, which were further intensified by the additional loss of tin function. The results of this study suggest that the K158N mutation in humans is likely to be pathogenic because of its dual effect on DNA-binding deficiency and reduced interaction with a cardiac cofactor. Furthermore, the manifestation of cardiac abnormalities associated with this mutation may occur during later stages of development or adulthood [42]. Zhu *et al.* (2017) utilized a Drosophila melanogaster model and high-throughput *in vivo* functional validation of candidate CHD genes (Table 5) [43].

Drosophila genetics provide a unique resource for studying human diseases that are unavailable in other models. However, the use of Drosophila as a CVD model poses several challenges. Unlike humans, flies have an open circulatory system and only one cardiac chamber functions like the heart. The myocardium receives oxygen through diffusion rather than through the coronary arteries. Additionally, ultra-structural analysis showed that myocytes have perforated Z-discs that allow supra-contractile characteristics that almost completely obliterate the heart chamber during systole. Despite these drawbacks, Drosophila can still be used for extensive genetic screening to understand heart development during embryogenesis and investigate cardiac abnormalities in adults [32].

## Zebrafish:

The zebrafish, scientifically known as Danio rerio, is a small tropical fish belonging to the minnow family Cyprinidae, originally found in Southeast Asia. Zebrafish and mammalian hearts retain the atria, ventricles, cardiac valves, and the cardiac conduction system. These traits help to identify zebrafish cardiovascular mutations and provide insights into human cardiovascular illnesses [44]. Zebrafish, as a vertebrate model, has gained significant popularity in the scientific community to investigate gene function and understand the underlying mechanisms of human genetic illnesses. The increased level of gene conservation has resulted in the increased utilization of zebrafish as an experimental model for studying human diseases. Despite its seemingly straightforward nature, the zebrafish heart demonstrates physiological characteristics comparable to those of the human heart, such as heart rate, contractile dynamics, and action potential [45]. A wide range of cardiovascular mutant phenotypes, including CHDs, have been identified in zebrafish. Moreover, several tools, including morpholinos, TILLING, TALEN, and zinc finger nucleases, have been developed to perturb specific genes of interest (reverse genetics), and subsequently used to model candidate CHD genes [46]. Zebrafish are particularly sensitive to small-molecule treatments and are thus suitable for chemical genetic studies and screening to identify additional cellular and molecular pathways that may regulate cardiovascular development. Through precise genome editing using single-stranded oligodeoxynucleotides, researchers have introduced the human PBX3 p.A136V variant into zebrafish pbx4 using CRISPR-Cas9 genome editing [46]. This study was performed to investigate whether this variant, which is more common in patients with CHD, acts as a genetic modifier in zebrafish heart development. The results showed that the pbx4 p.A131V variant could enhance myocardial morphogenesis defects caused by loss of hand2, a cardiac specification factor. These findings suggest that the pbx4 p.A131V allele may be a genetic modifier of the heart [46].

An additional investigation using a zebrafish model confirmed the role of a rare causative gene in congenital cardiomyopathy, which leads to a fatal restrictive phenotype [47]. This study used whole-exome sequencing and linkage analysis to investigate the genetic underpinnings of a newly characterized cardiac disorder in a Caucasian family. The family consisted of both unaffected and affected individuals, including a pair of twins. Researchers identified two genetic variations in KIF20A and conducted experiments using zebrafish embryos to investigate the effects of reducing KIF20A expression through MO-mediated knockdown. The results showed that the zebrafish embryos with reduced KIF20A expression exhibited a progressive cardiac phenotype characterized by red blood cells near the atrium, increased heart rate, and cardiac edema suggesting that KIF20A plays an important role in heart development and function [47]. Despite these advances, the use of zebrafish as a disease model has several limitations. The lack of cross-reactivity between mammalian and zebrafish antibodies limits the use of zebrafish as a model organism in protein biochemistry. Duplicated genes exhibit sub-functionalization, which complicates genetic analysis but allows for the study of several gene functions using mutants. The lack of embryonic stem cells for reverse genetic methods, such as knockout strain creation, has slowed scientific research on this organism [48].

## *In vitro* models:

## Induced pluripotent stem cells:

Induced pluripotent stem cells (iPSCs) can be derived from adult somatic cells by forced reprogramming to differentiate into almost all cell types [49]. Using patient-derived iPSCs offers a distinctive opportunity to investigate the genetic underpinnings of CHD as these cells maintain the complete genetic repertoire of the corresponding affected individuals. The integration of CRISPR/Cas9 genome editing, single-cell genomics, and cardiac organoid engineering techniques with iPSCs could serve as a valuable addition to existing mouse genetic models of CHD. Cardiomyocytes (CMs), vascular smooth muscle cells (SMCs), and endothelial/endocardial cells (ECs) derived from iPSCs can be used as human iPSC models of CHD [38]. Wang *et al.* used CMs produced from iPSC-CMs obtained from individuals with Barth syndrome to characterize many metabolic, structural, and functional irregularities linked to TAZ mutations. The data presented in this study suggest that the overproduction of reactive oxygen species (ROS) plays a role in the development of sarcomere disarray and decreases contractile stress generation in Barth syndrome (BTHS) iPSC-CMs. The involvement of ROS in CM development, sarcomerogenesis and contractility is known [50]. Patient-specific iPSC-CMs generated from patients with left ventricular non-compaction (LVNC) carrying a mutation in the cardiac transcription factor TBX20 are associated with perturbed transforming growth factor beta (TGF-β) signaling and a pathological LVNC phenotype at the single-cell level. In this study, TBX20 mutation was a probable causative agent of LVNC [51]. In 2019, Gifford *et al.* used human iPSCs to learn about CHD, especially to validate MKL2, MYH7, and NKX2-5 genes. Data revealed that NKX2-5 variations have been identified as a genetic modifier in cases of LVNC cardiomyopathy, where the age at which symptoms manifest might range from childhood to the incidental discovery of asymptomatic cases in adults, whereas in hypoplastic left heart syndrome (HLHS) patients, NOTCH1 gene mutations have been identified in iPSCs derived from these patients [52]. A set of differentially expressed genes (DEGs) in HLHS was significantly enriched in these heart failure coordinators. Notably, the mitochondrial components in all HLHS iPSC-CMs were reduced compared to those in control iPSC-CMs [53]. These findings can help us to understand CHD, as HLHS is a severe form of CHD. Kathiriya *et al.* recently generated TBX5 knockout human iPSC lines with heterozygous and homozygous mutations. Single-cell RNA sequencing and gene regulatory network analysis revealed that TBX5 haploinsufficiency alters the expression of CHD-related genes and reduced TBX5 disruption of gene regulatory networks in human iPSC-CMs. The abnormal genetic interaction between Tbx5 and Mef2c causes ventricular septation defects in transgenic mice with reduced Tbx5 dosage [54]. The current state of pluripotent stem cell-derived cardiomyocytes (PSC-CMs) indicates that using CMs sourced from adult organisms such as humans or rats could yield more accurate and relevant data for research. However, the use of adult CMs in *In vitro* studies remains challenging. When cultured under standard conditions, isolated primary adult CMs either die or lose their maturation characteristics rapidly. This loss of maturity results in diminished electrophysiological properties, decreased contractile function, and departure from the typical adult cellular structure, including loss of T-tubules within a short timeframe. A drawback of employing tissue engineering techniques is that they are less accessible, require specialized engineering skills, take a significant amount of time (over a month to establish), and create difficulties for subsequent analyses such as imaging thick tissue or extracting CMs from their complex 3D environment for certain tests. Moreover, implementing these methods for potential cell therapy applications presents scalability challenges [54]. Recent studies have demonstrated that iPSCs exhibit distinct DNA methylation patterns, indicating an imperfect reprogramming state. The potential ramifications of this phenomenon, known as "epigenetic memory", are yet to be fully understood. Recent studies have suggested that the origin of iPSCs influences their ability to differentiate. Although hiPSCs often exhibit comparable efficiency to hESCs in differentiating into specific lineages, there are instances where their pluripotent differentiation capacity is inadequate, which may be attributable to epigenetic constraints [55]. Furthermore, it should be noted that iPSC-CMs are often cultivated in a 2D format, which deviates from the 3D architecture of the human cardiac tissue. Patient iPSC-derived cardiac organoids have the potential to serve as effective 3D alternatives for studying the human heart [56].

## Human Pluripotent Stem Cells:

Human pluripotent stem cells (hPSCs) are obtained from embryos, embryonic stem cells (hESC), and iPSC. These cells can differentiate into cardiovascular cells [57]. The correlation between TCTN3 (RefSeq NM_015631.5)/LTBP2 (RefSeq NM_000428.2) mutation and the clinical phenotype of the patient was verified. Chen *et al.* established an hPSC model with point mutations using CRISPR/Cas9-mediated genome engineering [58]. LTBP2 mutation was found to cause changes in the rhythm development of CMs. In contrast, the group hPSCs-CM-TCTN3mutaion showed a significantly lower rate and weaker contraction force. These results suggest that mutations in LTBP2 and TCTN3 affect the early development of CMs, which affects the cardiac rhythm and contraction [58]. This investigation proved that mutations in LTBP2 and TCTN3 may serve as possible pathogenic factors in cases of complex CHD accompanied by polydactyly. These mutations have been linked to alterations in cellular processes, which can potentially affect heart development. Moreover, this study suggests that TBX5 mutations may not be present in cases of severe CHD associated with polydactyly [58].

Naive human cells produced by GSK3β, ROCK, BRAF, MEK, and SRC kinase inhibitors exhibit recurrent chromosomal aberrations [59]. Furthermore, naive hESCs exhibit a higher number of single-nucleotide variants (SNVs) than their primed counterparts. This phenomenon may occur because the DNA damage and repair mechanisms may be downregulated. Further research is necessary to comprehensively understand this issue. An additional issue with naive hPSCs is the global hypomethylation. After undergoing redifferentiation and returning to the primed state, most of the genomic regions underwent remethylation. In contrast, this does not hold for imprinted genes. Most imprinted patterns were erased in primed cells. Abnormal imprinting can impede the therapeutic use of naive human pluripotent stem cells [60]. Although hiPSCs exhibit comparable efficiency in differentiating into particular lineages as hESCs, there are instances in which hiPSCs display partial pluripotent differentiation capacity. This phenomenon can be attributed to epigenetic barriers [55].

## CRISPR/Cas9:

The use of CRISPR/Cas9 for direct mutagenesis is progressively improving and has the potential to aid in explicating genomic variations in the future [61]. It is essential to acknowledge that the CRISPR/Cas9 system has successfully targeted embryos of several mammalian species, including rats and monkeys, as well as non-mammalian organisms, such as Drosophila and zebrafish. CRISPR/Cas9 facilitates the creation of isogenic cell lines with high efficiency and simplicity. These cell lines possess the desired DNA sequence variation, eliminating potential confounding factors such as genetic background and epigenetic memory. CRISPR/Cas9 technology has demonstrated its efficacy and utility in facilitating gene knockout (KO) or knock-in in human cells [62]. CRISPR-Cas technology offers potential avenues for addressing hereditary CVD by correcting pathogenic mutations in the patient's DNA. SpCas9 and SaCas9, the most commonly used CAS proteins, have been extensively employed for CVD modeling and therapeutic applications *In vitro* and *in vivo* [63]. The main CHD-causing genes that were discovered or validated using CRISPR/Cas9 are listed in Table 6 [64]. Regrettably, certain constraints persist in CRISPR-Cas systems, which require resolution. These include the possibility of off-target effects, restricted genome-targeting scope due to protospacer-adjacent motif sequences, and suboptimal efficiency and specificity. Consequently, numerous research teams have endeavored to enhance this technology [65].

## Conclusion:

**Figure 2:**
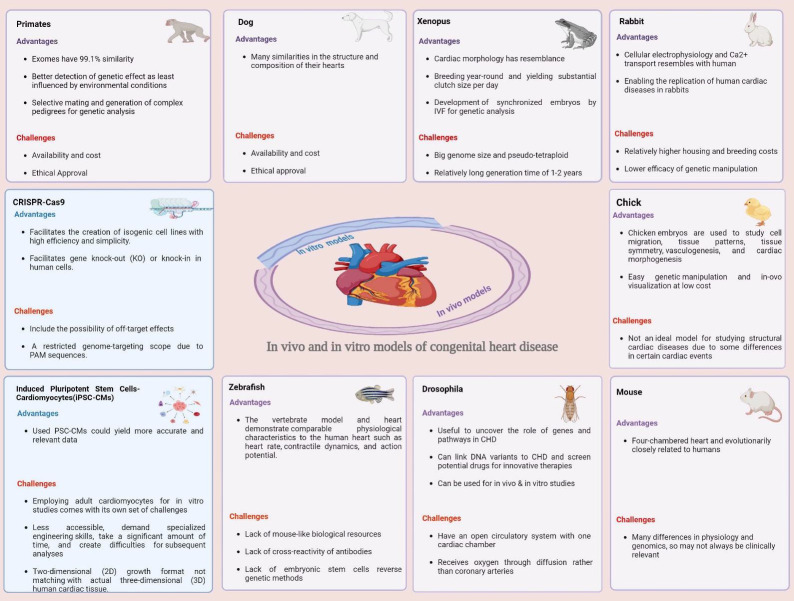
Advantages and disadvantages of congenital heart disease models.

Advances in epidemiological research have led to a significant increase in the global prevalence of CHD, whereas genetic studies have shed light on various genetic abnormalities associated with different types of CHD. Therefore, understanding the genetics of CHD is crucial to improve its management and treatment. Studies on CHD genes have encompassed several models and methods. Animal models, both genetically engineered and naturally occurring, have played a significant role in elucidating the genetic basis of CHD. These models, including primates, canines, frogs, rabbits, chicks, mice, Drosophila, and zebrafish, have provided insights into the molecular mechanisms of cardiac development and effects of genetic mutations. Primates offer a high degree of genetic similarity to humans; however, their limited availability and high costs have limited their widespread use. Canine dogs have a cardiac structure comparable to that of humans; however, their cost is significant. Xenopus frogs are a practical model owing to their abundant embryos, affordability, and genetic manipulability. However, pseudotetraploidy in X. laevis and the functional redundancy among genes pose challenges. Rabbits have great potential as CHD models because of their similar cellular electrophysiology to humans; however, limitations in genetic manipulation and reproductive rates exist. Chickens offer valuable insights owing to their ease of manipulation and low cost, but differences in certain cardiac events compared to humans exist. Mice with four-chambered hearts and extensive genetic resources are a promising model. However, variations in physiology and genomics have also been reported. Fruit flies share genetic parallels with humans; however, differences in cardiac structure and open circulatory systems present hurdles. Zebrafish, with their genetic conservation, exhibit physiological similarities to the human heart, but face challenges such as a scarcity of cross-reactivity with mammalian antibodies and gene duplication. Recent advancements in induced iPSCs, hPSCs, and CRISPR/Cas9 have significantly affected this field. Each model has distinct advantages and disadvantages. iPSCs maintain the genetic profiles of affected individuals, but are limited to 2D cell culture and genomic stability concerns. hPSCs can differentiate into cardiovascular cells, raising concerns regarding their genomic stability and imprinting loss. CRISPR-Cas9 technology is promising for correcting pathogenic mutations; however, off-target effects remain an issue. The advantages and disadvantages of this method are summarized in Figure 2. The choice of method or model for CHD gene research is determined by the specific research goals, available resources, and ethical considerations. Researchers must carefully evaluate these advantages and disadvantages to select the most suitable approach for their studies. It is important to recognize that there is no ideal animal model for the human cardiovascular system and relying on only one animal model to address all issues is not advisable. Future research should embrace interdisciplinary approaches to untangle the complex genetic landscape of CHD, ultimately leading to the development of more effective diagnostic tools and therapeutic interventions.

## Figures and Tables

**Table 1:** Genes Associated with non-syndromic CHD

**Genes associated with non-syndromic congenital heart disease**	
**Gene**	**Cardiovascular Defect**
CITED2	Atrial septal defect, ventricular septal defect
GATA4	Atrial septal defect, ventricular septal defect, atrioventricular septal defect, PS, Tetralogy of Fallot
GATA5	Atrial septal defect, ventricular septal defect, double outlet right ventricle, Tetralogy of Fallot, bicuspid aortic valve
GATA6	Percutaneous transluminal angioplasty, Tetralogy of Fallot
HAND1	atrioventricular septal defect, double outlet right ventricle, hypoplastic left heart syndrome, atrial septal defect, ventricular septal defect
HAND2	Tetralogy of Fallot, left ventricular noncompaction cardiomyopathy, ventricular septal defect.
JARID2	Left-sided lesions
MED13L	Transposition of the great arteries
NR2F2	Atrioventricular septal defect, aortic stenosis, coarctation of the aorta, ventricular septal defect, hypoplastic left heart syndrome, tetralogy of Fallot
NKX2-5	Atrial septal defect, atrioventricular conduction delay, tetralogy of Fallot, hypoplastic left heart syndrome, ventricular septal defect
NKX2-6	Percutaneous transluminal angioplasty
TBX1	Double outlet right ventricle, tetralogy of Fallot, interrupted aortic arch, percutaneous transluminal angioplasty, ventricular septal defect.
TBX5	atrioventricular septal defect, tetralogy of Fallot, bicuspid aortic valve, coarctation of the aorta, atrial septal defect, ventricular septal defect
TBX20	Atrial septal defect, ventricular septal defect, mitral stenosis, dilated cardiomyopathy
MEF2C	double outlet right ventricle
NFATC1	Tricuspid atresia, atrioventricular septal defect
ZFPM2/FOG2	Tetralogy of Fallot, double outlet right ventricle
ACVR1/ALK2	Atrioventricular septal defect
CFC1	Transposition of the great arteries, double outlet right ventricle
CRELD1	Atrial septal defect, atrioventricular septal defect
FOXH1	Tetralogy of Fallot, transposition of the great arteries, ventricular septal defect
GDF1	Atrial septal defect, double outlet right ventricle, transposition of the great arteries, tetralogy of Fallot
GJA1	Hypoplastic left heart syndrome
HEY2	Atrioventricular septal defect
JAG1	Tetralogy of Fallot, PS
NODAL	Transposition of the great arteries, double outlet right ventricle, tetralogy of Fallot, ventricular septal defect
NOTCH1	Bicuspid aortic valve, aortic stenosis, hypoplastic left heart syndrome, tetralogy of Fallot, PS, atrial septal defect, ventricular septal defect, coarctation of the aorta, double outlet right ventricle
PDGFRA	Total anomalous pulmonary venous return
SMAD6	Bicuspid aortic valve, coarctation of the aorta, aortic stenosis
TAB2	Bicuspid aortic valve, aortic stenosis, tetralogy of Fallot
VEGFA	Tetralogy of Fallot, patent ductus arteriosus, aortic stenosis, bicuspid aortic valve, coarctation of the aorta, interrupted aortic arch, ventricular septal defect.
ACTC1	Atrial septal defect, hypertrophic cardiomyopathy, dilated cardiomyopathy, left ventricular noncompaction cardiomyopathy.
DCHS1	Mitral valves prolapse
ELN	Supravalvular aortic stenosis
MYH6	Atrial septal defect, hypertrophic cardiomyopathy, dilated cardiomyopathy
MYH7	Ebstein's anomaly, left ventricular noncompaction cardiomyopathy, hypertrophic cardiomyopathy, dilated cardiomyopathy.
MYH11	Patent ductus arteriosus, thoracic aortic aneurysm

**Table 2:** Genes Associated with syndromic CHD

**Genes associated with syndromic congenital heart disease**	
**Gene**	**Cardiovascular Defect**
TBX1	DiGeorge syndrome
ELN	Williams-Beuren syndrome
ETS1	Jacobsen syndrome
FLI1	
JAG1	Alagille syndrome
NOTCH2	
TFAP2B	Char syndrome
CHD7	CHARGE syndrome
HRAS	Costello syndrome
EVC	Ellis-van Creveld syndrome
EVC2	
TBX5	Holt-Oram syndrome
KMT2D	Kabuki syndrome
KDM6A	
PTPN11	Noonan Syndrome
SOS1	
RAF1	
KRAS	
NRAS	
RIT1	
SHOC2	
SOS2	
BRAF	

**Table 3:** Xenopus models of human CHD

**Gene**	**Xenopus Model**	**Cardiovascular Phenotype**
*Shp*2	Atrial septal defects, ventricular septal defects, pulmonary stenosis, hypertrophic cardiomyopathy	Infused heart fields, loss of cardiac cells
*Zic*3	Cardiac looping defects, atrial septal defects, ventricular septal defects, transposition of the great arteries, double outlet right	Abnormal cardiac looping
*Nkx*2.5	Atrial septal defects, cardiac conduction system defects	Enlarged heart
*gata*4	Loss of Function	Looping defects
*nkx*2-5	Gain of Function	Cardiac conduction defects, atrial septal defect
*pitx*2	Gain of Function, Loss of Function	Looping defects and atrial septal defects
*chd*7	Gain of Function, Loss of Function	Neural crest migration and cardiac outflow tract defects
*tbx*1	Gain of Function	Looping defects
*tbx*5	Gain of Function, Loss of Function	Looping defects, reduced cardiomyocytes
*ets*1	Loss of Function	Cardiac outflow tract and aortic arch formation defects
*mctp*2	Gain of Function, Loss of Function	Looping defects, cardiac outflow tract defects
*tbx*20	Loss of Function	Looping defects, reduced cardiomyocytes

**Table 4:** Summarizes the mouse models of CHD

**Gene**	**Human CHD phenotype**	**CHD-Associated Syndrome**	**Murine Genotype**	**Murine Phenotype**
ACVR1 (ALK2)	Atrioventricular septal defect	NA	*Alk2^fl/-^; Tie2-Cre*	Atrioventricular septal defect, ventricular septal defect.
CITED2	Atrial septal defect, ventricular septal defect.	NA	*Cited2^-/-^*	Atrial septal defect, ventricular septal defect, double-outlet right ventricle, tricuspid atresia
CREBBP	Atrial septal defect, ventricular septal defect, coarctation of the aorta, pulmonic stenosis, bicuspid aortic valve	Rubinstein-Taybi syndrome	*CBP^+/-^*	Atrial septal defect, ventricular septal defect, bicuspid aortic valve
EP300	Atrial septal defect, ventricular septal defect, coarctation of the aorta, pulmonic stenosis, bicuspid aortic valve	Rubinstein-Taybi syndrome	*EP300^+/AS^*	Atrial septal defect, ventricular septal defect
GATA4	Atrial septal defect, pulmonic stenosis, tetralogy of Fallot, ventricular septal defect, Atrioventricular septal defect	NA	*Gata4^Δex2/wt^*	Atrial septal defect, ventricular septal defect, Atrioventricular septal defect
			*Gata4^G295Ski/wt^*	Atrial septal defect, aortic stenosis, pulmonic stenosis
KMT2D	Aortic stenosis, coarctation of the aorta, Atrial septal defect, ventricular septal defect, bicuspid aortic valve, hypoplastic left heart syndrome, tetralogy of Fallot	Kabuki syndrome	*Kmt2d^fl/fl^; Mef2c-AHF-Cre*	Ventricular septal defect
NIPBL	Atrial septal defect, ventricular septal defect, pulmonic stenosis	Comelia de Lange syndrome	*Nipbl^+/-^*	Atrial septal defect, ventricular septal defect
NKX2-5	Atrial septal defect, atrioventricular conduction delay, tetralogy of Fallot, VSD, hypoplastic left heart syndrome	NA	*Nkx2 5^+/-^*	Atrial septal defect, patent foramen ovale, ventricular septal defect, Atrioventricular septal defect, bicuspid aortic valve, AS
			*Nkx2.5^+/R52G^*	Atrial septal defect, ventricular septal defect, Atrioventricular septal defect, Ebstein's anomaly, atrioventricular block, tricuspid valve atresia
			*Nkx2.5^R141C/+^*	Atrial septal defect, atrioventricular block, ventricular septal defect
PTPN11	Pulmonic stenosis, Atrioventricular septal defect, coarctation of the aorta, Atrial septal defect, ventricular septal defect, TOF, left ventricular outflow tract obstruction.	Noonan syndrome	*Ptpn11^D61G/+^*	Atrial septal defect, Atrioventricular septal defect, double-outlet right ventricle
SHOC2	Pulmonic stenosis, Atrioventricular septal defect, coarctation of the aorta, Atrial septal defect, ventricular septal defect, tetralogy of Fallot	Noonan syndrome	*Sur-8^Δ/fl^; Tie2-Cre*	ventricular septal defect, double-outlet right ventricle, transposition of great arteries
TBX5	Atrial septal defect, ventricular septal defect	Holt-Oram syndrome	*Tbx5^del/+^*	Atrial septal defect, atrioventricular block, ventricular septal defect
			*Tbx5^flox/flox^; Tie2-Cre*	Atrial septal defect, patent foramen ovale
	Atrial septal defect, ventricular septal defect, Atrioventricular septal defect, tetralogy of Fallot	Down syndrome	*Tc1*	Ventricular septal defect, atrioventricular septal defect
			*Dp(10)1Yey/+;Dp(16)1Yey/+;Dp(17)1Yey/+*	Ventricular septal defect, Atrioventricular septal defect
			*Dp1Tyb*	Ventricular septal defect, Atrioventricular septal defect, double-outlet right ventricle
			*Dp3Tyb*	
DCHS1	Mitral valves prolapse	NA	*Dchs1^+/-^*	Mitral valves prolapse
GATA5	Bicuspid aortic valve	NA	*Gata5^-/-^*	Bicuspid aortic valve, aortic valve stenosis
			*Gata5^fl/fl^; Tie2-Cre*	
GATA6	TA, Atrial septal defect, tetralogy of Fallot, bicuspid aortic valve	NA	*Gata6^+/-^*	Bicuspid aortic valve
			*Gata6^wt/fl^; Isl1-Cre*	
MATR3	Bicuspid aortic valve, coarctation of the aorta, patent ductus arteriosus	NA	*Matr3^+/-^*	Bicuspid aortic valve, coarctation of the aorta, patent ductus arteriosus, ventricular septal defect, double-outlet right ventricle
NOTCH1	Bicuspid aortic valve, aortic valve stenosis, hypoplastic left heart syndrome, tetralogy of Fallot, pulmonic stenosis, calcific aortic valve disease	NA	*Notch1^+/-^*	Bicuspid aortic valve, calcific aortic valve disease, aortic aneurysm
			*Notch1^fl/fl^; Nfatc1-enCre*	Bicuspid aortic valve
			*Notch1^+/-^ mTR^G2^*	Calcific aortic valve disease, aortic valve stenosis
			*Nos3^-/-^; Notch1^+/-^*	Bicuspid aortic valve, aortic valve stenosis, AR, calcific aortic valve disease, tetralogy of Fallot
SMAD6	Bicuspid aortic valve, aortic valve stenosis, coarctation of the aorta	NA	*Smad6^-/-^*	Cardiac valve hyperplasia
CHD7	tetralogy of Fallot, double-outlet right ventricle, ventricular septal defect, Atrial septal defect, truncus arteriosus, pulmonic stenosis, aortic valve stenosis, MS, tricuspid valve stenosis	CHARGE syndrome	*Chd7^+/-^*	Interrupted aortic arch, aortic arch defects
CRKL	Tetralogy of Fallot, truncus arteriosus, interrupted aortic arch, ventricular septal defect, aortic arch defects	22q11 deletion syndrome	*Crkol^-/-^*	Interrupted aortic arch, ventricular septal defect, overriding aorta, double-outlet right ventricle
FOXC1	Tetralogy of Fallot	NA	*Foxc1^-/-^*	Coarctation of aorta, semilunar valve dysplasia, interrupted aortic arch, ventricular septal defect.
FOXC2	Tetralogy of Fallot	NA	*Foxc2^-/-^*	Interrupted aortic arch, ventricular septal defect
FOXH1	Tetralogy of Fallot, ventricular septal defect	NA	*Foxh1^C/-^*	Right isomerism, Atrial septal defect, ventricular septal defect, transposition of great arteries, double-outlet right ventricle
JAG1	Tetralogy of Fallot, pulmonic stenosis, atrial septal defect, ventricular septal defect	Allagille syndrome	*Jag1^fl/fl^; Islet1-Cre Jag1^fl/fl^; Mef2c-AHF-Cre*	Double-outlet right ventricle, pulmonic stenosis, truncus arteriosus, atrial septal defect, ventricular septal defect, aortic arch defects
TBX1	Tetralogy of Fallot, truncus arteriosus, interrupted aortic arch, ventricular septal defect, aortic arch defects	22q11 deletion syndrome	*Df1/+*	Aortic arch defects, ventricular septal defect
			*Tbx1^Neo2/Neo^*	Tetralogy of Fallot, truncus arteriosus, double-outlet right ventricle, interrupted aortic arch, ventricular septal defect, aortic arch defects.
			*Tbx1^neo/neo^*	Truncus arteriosus, interrupted aortic arch, ventricular septal defect, aortic arch defects.
			*Tbx1^+/-^*	Interrupted aortic arch, aortic arch defects
ZFPM2(FOG2)	Tetralogy of Fallot, double-outlet right ventricle	NA	*Fog2^-/-^*	Tetralogy of Fallot, atrial septal defect, ventricular septal defect
ELN	Supravalvular aortic stenosis	Williams-Beuren syndrome	*Eln^+/-^*	Supravalvular aortic stenosis
FBN1	Bicuspid aortic valve, aortic valve regurgitation, mitral valve prolapses, aortic aneurysm, aortic dissection	Marfan syndrome	*Fbn1^C1039G/+^*	Mitral valve prolapses, aortic aneurysm

**Table 5:** Validated CHD-associated genes and their Drosophila analogs

**Human Gene**	***Drosophila*** **Homolog**	**Type of Mutation**	**Mutated AA**	**Gene ID#**
LIG1	DNA-ligI	Nonsense	Y765X	34564
				106463
NCKAP1	Hem	Nonsense	E1057X	29406
				41688
				103380
GTPBP4	Non1	Nonsense	K332X	31117
				100270
OS9	CG6766	Frameshift	T158	42924
FTSJ3	CG8939	Frameshift	786/847	40726
SERPINH1	Spn28Dc	Nonsense	R415X	34381
LAMC1	LanB2	Missense	G170E	104013
				42560
TLN1	Rhea	Missense	L684V	32999
				33913
OBSCN	Unc-89	Missense	F5295S	31538
			T4421M	31539
LAMA5	LanA	Missense	C1625Y	28071
				18873
GANAB	CG14476	Missense	N171S	34334
				48375
DST	Shot	Missense	K2653I	28336
			G2936D	41858
EIF3H	eIF-3P40	Missense	H109R	36086
				106189
FYCO1	Rbpn-5	Missense	E1286K	52996
RNF44	Mura	Missense	R421Q	35236
TSHZ1	Tio	Missense	Q288R	35812
	Tsh			28022
RUFY2	CG31064	Missense	P621L	60496
EFHD2	Swip-1	Missense	A230V	31585
PHIP	BRWD3	Missense	S674C	33421
C11orf9	CG3328	Missense	F387S	55211
CPSF1	Cpsf160	Missense	N29K	55698
LZTR1	CG3711	Missense	G248R	33422
GTPBP1	Dgp-1	Missense	E291K	27490
				27493
KIAA0196	CG12272	Missense	V167D	51906
SMAD4	Med	Missense	I500V	31928
KPNA1	Kap-alpha1	Missense	P350S	27523
DHX38	l(1)G0007	Missense	G332D	57153
MINK1	Msn	Missense	R299C	28791
				42518
				101517
NTM	CG31646	Frameshift	204/344	28654
ODZ4	Ten-a	Missense	R1444K	29439
COL4A3BP	Cert	Missense	G131D	60080
PAPSS1	Papss	Missense	T399S	60079
KCNH6	Sei	Missense	T274M	31682
SSH2	Ssh	Missense	V108L	38948
XRCC5	Ku80	Missense	K238Q	27710
NAA16	Nat1	Missense	R70C	32357
DTNA	Dyb	Missense	P295S	32935
ITGA7	Mew	Missense	R279W	44553
PIK3CD	Pi3K92E	Missense	L347V	61182
NR6A1	Hr4	Missense	C120R	54803
BICD1	BicD	Missense	D760E	35405
ALPL	CG5656	Missense	A102T	58334
	CG10827			57526
RDH5	Sni	Missense	R280S	31978
FGFR4	Htl	Missense	D297N	58289
GRM8	Mtt	Missense	N778S	44076
TTN	Bt	Missense	T4852N	31546
PFKM	Pfk	Missense	A522G	34336
LAMB2	LanB1	Missense	R1661W	42616
NUCB1	NUCB1	Missense	R189C	44019
STAB1	CG11377	Missense	A1102V	51741
CPD	Svr	Missense	P425R	44487
LRPPRC	Bsf	Missense	D486N	34550
DSG2	CadN2	Missense	L499Q	38207
MYEF2	Rump	Missense	I264V	42665
AP3B1	Rb	Missense	E771K	28668
NUP62	Nup62	Missense	Q70R	52927
TOMM40L	Tomboy40	Missense	S171I	29573
	Tom40			26005
MAP2K7	Hep	Missense	V409I	28710
ELMO2	Ced-12	Missense	N332S	36097
NOP2	CG8545	Missense	I351V	56998
PRPF4B	CG7028	Missense	E14Q	55640
GRIP2	Grip	Missense	T954M	40930
CDH23	Ds	Missense	R1136C	28008
APLP1	Appl	Missense	R330C	39013
MPI	CG8417	Missense	A38V	34379
TFIP11	Sip1	Missense	M432T	56933
TARS2	Aats-thr	Missense	P155R	42902
NCAPD3	Cap-D3	Missense	A1041V	61979
NFATC2	NFAT	Missense	D584A	51422
DDX10	CG5800	Missense	V427L	43206
ITPR3	Itp-r83A	Missense	R1027H	51686
VPS13C	Vps13	Missense	T423A	42625
NEURL2	CG3894	Missense	S92T	42618
WIBG	Wibg	Missense	G203V	36096
TWF2	Twf	Missense	E185Q	57375
BACH2	Cnc	Missense	T803A	32863
PPWD1	CG3511	Missense	I190V	50597
PKN3	Pkn	Missense	R255Q	57804
CREB5	Atf-2	Missense	T236M	33379
HIVEP2	Shn	Missense	P123L	34689
SBNO2	CG3491	Missense	V78M	57556
LPHN3	Cirl	Missense	K1406R	34821
MASTL	Gwl	Missense	D537N	34525
CRB2	Crb	Missense	R1189Q	38903
PABPC4L	pAbp	Missense	K224Q	60473
C16orf48	CG11125	Missense	A192T	58164
FAN1	Sn	Missense	T905M	42615
USH1C	CG5921	Missense	R875K	61859
NCKAP5	CG42663	Missense	T1202I	54808
CHIC1	CG5938	Missense	R129H	55613
DDO	CG12338	Missense	A107V	57779
ALS2CL	CG7158	Missense	R129W	28533
UNC13C	Unc-13	Missense	R1182Q	29548
AIPL1	CG1847	Missense	E195K	44490
KCNJ15	Irk2	Missense	T77I	41981
ANKS1B	CG4393	Missense	A67V	58087
RAB11FIP4	Nuf	Missense	E138K	44035
DNAH9	Dhc93AB	Missense	R668W	51511
FABP2	Fabp	Missense	R11Q	34685
ABCA13	CG34120	Missense	E574Q	34596
GPR1	AstC-R2	Missense	A293S	36888
DMBX1	Repo	Missense	E140Q	50735
DNAJC5B	Csp	Missense	E22K	33645
DSC1	CadN	Missense	V550D	27503
KCNH5	Eag	Missense	N817S	31679
ASIC4	Ppk7	Missense	R593W	31878
PDCD1LG2	Tutl	Missense	S36N	54850
ABCB6	Hmt-1	Missense	A176G	53284
MLL2	Trx	Frameshift	S1722	28899
				36684
CUL3	Cul-3	Frameshift	I144	46685
				10762
CHD7	Kismet	Nonsense	Q1599X	31351
				35443
RNF20	Bre1	Nonsense	Q83X	34990
				17571
NAA15	Nat1	Frameshift	D335	25845
		Nonsense	S761X	31466
NF1	Nf1	Splice	Exon 6 (+4 bp)	25845
				31466
KDM5B	Lid	Splice	Exon 12 (+1 bp)	28944
KDM5A		Missense	R1508W	36652
HUWE1	CG8184	Missense	R3219C	36715
				26935
NUB1	CG5111	Missense	D310H	28642
	CG15445			28643
DAPK3	Drak	Missense	P193L	55904
SUPT5H	Spt5	Missense	E451D	34837
				106814
BCL9	Lgs	Missense	M1395K	37476
				41983
USP34	Puf	Missense	L432P	106192
				27517
SUV420H1	Hmt4-20	Missense	R143C	32892
				36639
RAB10	Rab10	Missense	N112S	26289
				101454
FBN2	Frac	Missense	D2191N	31578
MED20	MED20	Splice	Exon 2 (+2 bp)	34577
				52483
SMAD2	Smox	Splice	W244C	43138
		Missense		41670
WDR5	Wds	Missense	K7Q	32952
				60399
UBE2B	UbcD6	Missense	R8T	35476
				42631
USP44	Scny	Missense	E71D	40877
PTCH1	Ptc	Missense	R831Q	28795
				44612
SOS1	Sos	Missense	T266K	34833
				31597
PITX2	Ptx1	Missense	A47V	107785
				19830
LRP2	Mgl	Missense	E4372K	29324

**Table 6:** Applications of CRISPR-Cas9 technology in CHDs

**CHD Form**	**Genes**	**Mutations**	**Cardiac anomalies**	**Model system**	**Cas9 type**
DiGeorge syndrome	DGCR2	DGCR2 destroy	IAA	Mouse TT2 ES cell	NFL-hCas9; sgRNA exon4
			PTA		
	TBX1	Knockout	TOF	E14-Tg2a mESCs	Alt-R
			VSD		SpCas9
Barth syndrome	TAZ	328T>C	Dilated cardiomyopathy	Human induced pluripotent stem cell line	Cas9
Wolff-Parkinson-White	PRKAG2	H530R	Ventricular tachyarrhythmia	Mouse	Cas9
Duchenne muscular dystrophy	Dystrophin	Nonsense mutation (exon 23)	Dilated cardiomyopathy	Mouse, zygote	Cas9 mRNA
				Mouse	aav9-SaCas9
Holt-Oram syndrome	TBX5	zTbx5b knockout	Atrial septal defect, atrioventricular septal defect, progressive atrioventricular conduction disease	Zebrafish	Cas9 mRNA
					sgRNA
		243-1G>C			
		148-1G>C			
		S196ter, DGlu243Fter, R237W			
Heterotaxy syndrome	ZIC3	890G > T (C297F)	Double inlet left ventricle, double-outlet right ventricle, d-TGA, atrioventricular septal defect, single atrium, tricuspid atresia, transposition of the great arteries, pulmonary atresia, ventricular septal defect, patent ductus arteriosus, left superior vena cava	Zebrafish mutation	zCas9 mRNA
		680dup			
		842_843del			
		869del			
		1063G>T			
		1111A>C			
		1060+1G>A			
	DNAH10	12q24.31 3-duplicate		Zebrafish knockout	
	RNF115	1q21.1 1-deletion		Zebrafish knockout	
	CFC1	R78W, R112C, R189C, G174del1		Mouse, zebrafish	
Noonan syndrome	PTPN11	922A > G, c.923A > G (exon 8)	Pulmonary valve stenosis		
			Hypertrophic cardiomyopathy		
		exon 2,3,4,7,8, 13	Delayed psychomotor development	Induced pluripotent stem cells	Cas9
		T59A			
	LZTR1	Intronic			
	KRAS	458A > T			
	RAF1	S259T			
	SOS1	K170E			
Marfan syndrome	FBN1	4282 delC 7_8insTC 2192 delC	Aortic root dilation, aortic root dissection, mitral valve prolapse	Human embryo	BE3
		T7498C			
	FBLN4	1189G>A (exon 11)		Zebrafish	
	TGFBR2	W521R R528H R537P			
	TGFBR1	973+1G>A 806-2A>C (exon5)			
Non-syndromic	GATA4	G296S	Atrial septal defect, ventricular septal defect	Induced pluripotent stem cells	spCas9 (H840A)
	MyHC6	R443P	Hypoplastic left heart syndrome	Induced pluripotent stem cells	Cas9
	NKX2.5	A119S	Left ventricular noncompaction cardiomyopathy	Induced pluripotent stem cells	Cas9
	MYH7	L387F	Left ventricular noncompaction cardiomyopathy	Induced pluripotent stem cells	Cas9
	MKL2	Q670H	Left ventricular noncompaction cardiomyopathy	Induced pluripotent stem cells	Cas9
